# Eggshells as hosts of bacterial communities: An experimental test of the antimicrobial egg coloration hypothesis

**DOI:** 10.1002/ece3.3508

**Published:** 2017-10-16

**Authors:** Donald C. Dearborn, Symmantha M. Page, Miri Dainson, Mark E. Hauber, Daniel Hanley

**Affiliations:** ^1^ Department of Biology Bates College Lewiston ME USA; ^2^ College of Veterinary Medicine Midwestern University Glendale AZ USA; ^3^ Department of Animal Biology School of Integrative Biology University of Illinois Urbana‐Champaign IL USA; ^4^ Department of Biology Long Island University – Post Brookville NY USA

**Keywords:** 16S rRNA, antimicrobial, bacteria community, eggshell color, high‐throughput sequencing, photosensitization, protoporphyrin

## Abstract

Oviparous animals have evolved multiple defenses to prevent microbes from penetrating their eggs and causing embryo mortality. In birds, egg constituents such as lysozyme and antibodies defend against microbial infestation, but eggshell pigments might also impact survival of bacteria. If so, microbes could exert an important selective pressure on the evolution of eggshell coloration. In a previous lab experiment, eggshell protoporphyrin caused drastic mortality in cultures of Gram positive, but not Gram negative, bacteria when exposed to light. Here, we test this “photodynamic antimicrobial hypothesis” in a field experiment. In a paired experimental design, we placed sanitized brown, protoporphyrin‐rich chicken eggs alongside white eggs that lack protoporphyrin. We deployed eggs for 48 hr without incubation, as can occur between laying and incubation, when microbial infection risk is highest. Eggs were placed on the open ground exposed to sunlight and in dark underground storm‐petrel burrows. We predicted that the proportion of Gram‐positive bacteria on brown eggs should be lower when exposed to sunlight than when kept in the dark, but we expected no such difference for white eggs. Although our data revealed variation in bacterial community composition, the proportion of Gram‐positive bacteria on eggshells did not vary by egg color, and there was no interaction between egg color and location. Instead, Gram‐positive bacteria were proportionally more common on eggs on the ground than eggs in burrows. Overall, our experiment did not support the photodynamic antimicrobial hypothesis. The diverse range of avian egg colors is generated by just two pigments, but over 10 hypotheses have been proposed for the evolution of eggshell color. If our results are generalizable, eggshell protoporphyrin might not play a substantial role in defending eggs against microbes, which narrows the field of candidate hypotheses for the evolution of avian eggshell coloration.

## 1 INTRODUCTION

In oviparous organisms, the embryo develops while exposed to the external environment, including predators and diseases. In birds, which are exclusively oviparous, the eggshell surface and calcium carbonate matrix are host to a diverse bacterial fauna (Baggott & Graeme‐Cook, 2002; Grizard, Dini‐Andreote, Tieleman, & Salles, 2014; Kobayashi, Gutierrez, & Hatta, 1996), and trans‐shell penetration by some microbes can be lethal to the developing embryo (Cook, Beissinger, Toranzos, Rodriguez, & Arendt, 2005; Godard, Morgan Wilson, Frick, Siegel, & Bowers, 2007). Consequently, diverse antimicrobial barriers and defenses have evolved on and within the avian egg (D'Alba, Maia, Hauber, & Shawkey, 2016; D'alba & Shawkey, 2015). Those defenses that are interior to the egg include lysozyme, ovotransferrin, and other antimicrobial proteins in the albumen and the vitelline membrane (Guyot et al., 2016; Wellman‐Labadie, Picman, & Hincke, 2008). But the first line of antimicrobial defense is the eggshell, which provides a physical barrier of cuticular spheres (D'Alba et al., 2016), the shell matrix itself (Berrang, Cox, Frank, & Buhr, 1999), and an embedded set of lectin‐like proteins within the shell matrix (Wellman‐Labadie, Lakshminarayanan, & Hincke, 2008).

The survival of microbes on the surface and within the eggshell might also be impacted by eggshell chemistry, including its colorful pigmentation. The evolution of avian eggshell coloration has intrigued biologists for over a century (Swynnerton, 1916), with hypotheses for the origin and function of egg pigmentation rooted in predator–prey interaction, brood parasitism, thermal ecology, embryonic light exposure, and sexual selection (Hanley, Doucet, & Dearborn, 2010; Kilner, 2006; Maurer, Portugal, & Cassey, 2011). However, little attention has been paid to a possible selective pressure from microbes (Fargallo, López‐Rull, Mikšík, Eckhardt, & Peralta‐Sánchez, 2014). Despite the striking interspecific diversity in avian eggshell color, only two pigments seem to be involved: protoporphyrin IX, appearing brown, and biliverdin IXα, appearing blue–green (Hanley, Grim, Cassey, & Hauber, 2015). Of these two pigments, protoporphyrin has been shown to have light‐activated (i.e., photodynamic) antimicrobial defensive properties (Ishikawa et al., 2010).

Protoporphyrin's photodynamic antimicrobial defense appears to reduce or inhibit the proliferation of Gram‐positive bacteria (Ishikawa et al., 2010). This effect has been shown in a careful set of lab experiments (Ishikawa et al., 2010), using cultures of four commercially obtained bacterial strains. The experiments tested for antibacterial properties of eggshells from domestic chickens (*Gallus gallus domesticus*), comparing eggs that were solid brown, solid blue–green, and white, which contain, respectively, primarily protoporphyrin, biliverdin, and no pigments at all (Verdes et al., 2015). Exposure of bacteria to brown eggshells reduced the survival of Gram‐positive species (*Staphylococcus aureus*,* Bacillus cereus*) by more than two orders of magnitude, but no effect was seen on Gram‐negative bacteria (*Escherichia coli*,* Salmonella enteritidis*). Critically, the effect was observed only when illuminated with (artificial) light. These results are consistent with the hypothesis that Gram‐positive bacteria are susceptible to photosensitizers such as protoporphyrin. Follow‐up experiments on *S. aureus* cultures used purified pigments rather than intact eggshells, and found similar patterns—that protoporphyrin, but not biliverdin, caused light‐dependent reductions in the survival of Gram‐positive bacteria but not Gram‐negative bacteria (Ishikawa et al., 2010).

The results of these lab experiments on the antimicrobial properties of pigmented eggshells are striking, and this phenomenon could add an important novel dimension to considerations of the evolutionary origins and current functions of eggshell pigmentation and the resulting coloration (Lahti & Ardia, 2016). However, we do not know whether these findings are ecologically relevant, because no parallel data exist from field studies of bird eggs or from more complex bacterial communities.

**Figure 1:**
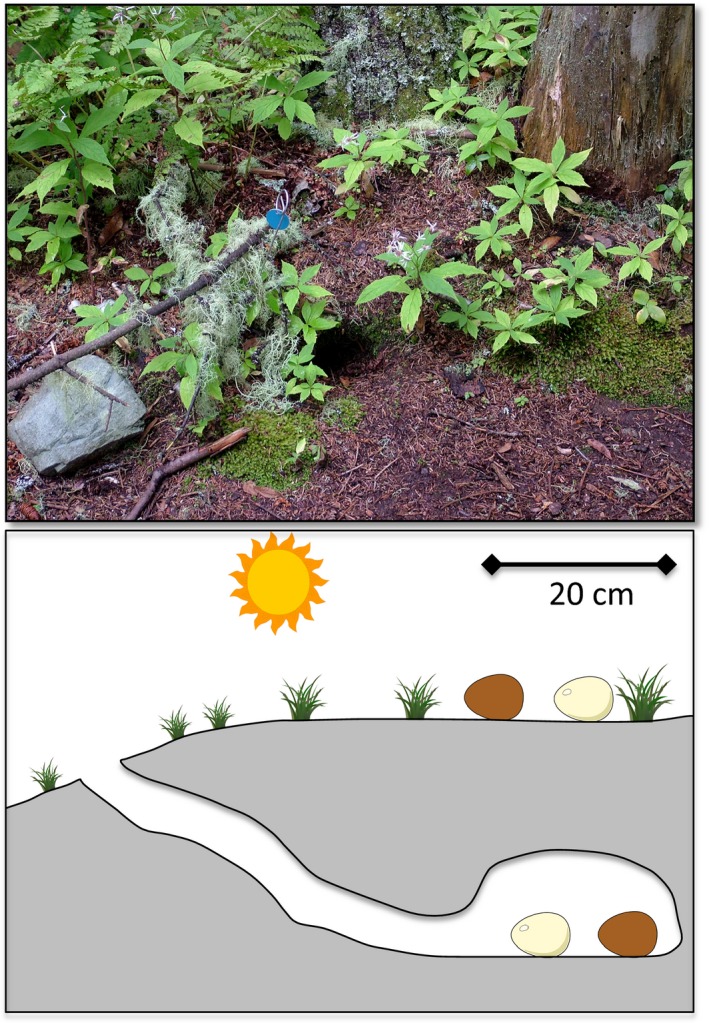
Top: Forest floor of the study site, showing an entrance to a storm‐petrel burrow to the right of the blue metal tag. Bottom: Experimental arrangement of each of 11 sets of four eggs. In each set of four, a brown egg and a white egg were placed side‐by‐side, separated by 4 cm, in an inactive storm‐petrel burrow and also on the ground surface above the burrow

Here, we report a field experiment testing for photodynamic antibacterial activity against Gram‐positive bacteria using high‐throughput sequencing data to characterize the diverse bacteria communities of avian eggshells. As in the earlier study (Ishikawa et al., 2010), we reduced other sources of variation using eggs with and without protoporphyrin from a single species, the domestic chicken. After sanitizing unincubated eggs, we deployed them for 48 hr in a natural field setting. This deployment simulates the period between laying and the onset of incubation, when eggs are unheated and exposed to different sets of microbes (Cook, Beissinger, Toranzos, & Arendt, 2005; Cook, Beissinger, Toranzos, Rodriguez et al., 2005) and ambient light regimes relative to the incubation period, due to the absence of parental incubation. In natural clutches, the duration of this period varies from less than one day to several days and can vary markedly within species (Hebert, 2002; Wang & Beissinger, 2009). The period of 48 hr used in our experiment would typify species or individuals that lay small clutches or that begin incubating well before clutch completion, but also would be experienced by particular eggs that are late in the laying sequence of larger clutches. Eggs were experimentally positioned in the environment in two types of locations that are part of the spectrum of avian nesting sites: on the open ground exposed to sunlight, and in dark underground burrows dug by Leach's Storm‐petrels, *Oceanodroma leucorhoa* (Figure 1). The antimicrobial hypothesis predicts that the percentage of an egg's bacteria that are Gram positive will be lower when exposed to sunlight than when kept underground in the dark, but only for brown eggs that contain protoporphyrin—that is, there should be an interaction between egg color and sunlight exposure with respect to Gram‐positive bacterial abundance.

To assess whether our methods were sufficient to detect biologically meaningful variation in bacteria community composition of avian eggshells, and to lay groundwork for a better understanding of natural microbe–eggshell interactions and the scale at which these interactions vary, we also characterize more broadly the composition of bacteria communities on different eggs, comparing across egg color, location, and date.

## 2 MATERIALS AND METHODS

### 2.1 Study site and sampling

Fieldwork was conducted during June 2014 in a Leach's Storm‐petrel breeding colony located at the Bowdoin Scientific Station on Kent Island, an 80‐ha island in the Bay of Fundy, New Brunswick, Canada (44.588N, 66.818W). The island sustains a mix of forested and open grassy areas. Common trees at the site of the experiment are American mountain ash (*Sorbus americana*), red spruce (*Picea rubens*), balsam fir (*Abies balsamea*), heartleaf birch (*Betula cordifolia*), and yellow birch (*B. alleghaniensis*), with a sparse groundcover of ferns, grasses, and raspberry (*Rubus spp*.). Typical storm‐petrel burrows at this study site are circa 60 cm long, culminating in a nest chamber that is 15 to 30 cm in diameter and sitting beneath 14 cm of overhead soil (Fricke et al. 2015; Figure 1). Soil comprising the floor of the nest chamber is typically quite wet, averaging over 3 g H_2_O/g dry mass (Fricke et al. 2015).

During the five‐day period of the different stages of our field experiment, the ambient above‐ground temperature on Kent Island ranged from 7 C to 19 C. Average humidity exceeded 90%, and on 18 June it rained 2.4 cm in the morning. Average cloud cover during the experiment was approximately 30%, and the time between each sunrise and sunset was 15 hr 34 min.

Using eggs from wild birds would typically require using two different species—one for brown eggs and one for white eggs—which would introduce an additional number of confounding variables. Thus, we used the artificially selected eggshell color polymorphism of a domesticated species, by commercially sourcing brown (*n* = 22) or white (*n* = 22) chicken eggs. We stored eggs at 4°C and then sanitized them through wiping the full surface with 70% ethanol (Mcdonnell & Russell, 1999) before deployment for 48 hr in the field.

Gloves were worn during all egg handling. At each of 11 sites, four eggs were deployed (*N* = 44 eggs) such that a brown egg and a white egg were placed 4 cm apart on the surface of the ground within 50 cm of an inactive storm‐petrel burrow, and another pair of brown and white eggs was deployed below ground in the center of that burrow's nest chamber (Figure 1). Eggs were deployed at six sites on 16 June 2014 and at five additional sites on 18 June 2014; the 11 sites were randomly divided between the two deployment dates. All eggs were deployed in the afternoon and retrieved 48 hr later.

To reduce the potential confound of having birds differentially interact with the eggs, we placed eggs in empty, unoccupied burrows that had not been used by breeding birds since at least the previous year, and we layered the burrow entrance with a lattice of fern stems to allow us to later confirm that indeed no bird had visited any of the test burrows during the 48 hr experiment. To prevent predation of above‐ground eggs by corvids and gulls while still allowing the penetration of sunlight, the eggs were covered by a wire mesh frame (20 cm diameter × 13 cm high) that was staked into the ground.

Upon collection, eggs were placed into ethanol‐sanitized containers using sterile gloves. We chose to extract DNA by shell crushing, because this approach has been shown to yield a more complete view of the diversity and community structure of eggshell bacteria compared to simply swabbing a sector of the shell (Grizard et al., 2014). Each eggshell was sectioned along its long axis with a sanitized Dremel rotary cutting tool, yielding one‐half that had been on the ground and another half that had been facing up. Yolk and albumin were discarded. Each half of the shell was put into its own sterile 50 ml conical tube and stored at −20°C. For extraction of pigments and DNA, frozen shell sections were individually pulverized with mortar and pestle which had been cleaned with 10% bleach and 70% ethanol and then autoclaved.

### 2.2 Protoporphyrin quantification

We randomly selected a subsample of 24 eggs (white *n* = 12, brown *n* = 12) for protoporphyrin and biliverdin analysis, following the methods detailed in Verdes et al. (2015). Briefly, we took 0.200 g of pulverized eggshell and used the ethylenediaminetetraacetic acid (EDTA) pigment extraction protocol (Gorchein, Lim, & Cassey, 2009; Verdes et al., 2015), ultimately resulting in 1 ml of dissolved sample in acetonitrile–acetic acid (4:1 v/v). Within 24 hr of sample preparation, the supernatants were measured for their UV absorbance in a Cary 300 UV‐Vis spectrophotometer. UV‐Vis spectrum readings were tracked from 250–700 nm, with measurements for biliverdin and protoporphyrin absorbance taken at 377 nm and 405 nm, respectively (Igic et al., 2010; Verdes et al., 2015). Samples were then analyzed for the presence of protoporphyrin and biliverdin on an Liquid Chromatography Mass Spectrometry (LCMS) system comprising an Agilent 1200 LC coupled to an Agilent 6340 ion trap MS. Samples (8 μl) were injected onto an Agilent Zorbax column (SB‐C8, 5 μmol/L, 2.1 × 50 mm) using a linear gradient of 5%–95% acetonitrile in water (0.1% formic acid) at a flow rate of 200 μl/min over 10 min. Protoporphyrin presence was indicated by a peak at 563 m/z and biliverdin presence indicated at 583 m/z (Verdes et al., 2015). When a pigment was detected, the concentration of samples was quantified using Beer–Lambert law (*A *= ε*bc*), where *A* is the absorbance at the specified wavelength, ε the molar extinction coefficient for the compound (protoporphyrin at 171000, and biliverdin at 56200), and *b* the path length of the sample (10 mm). Sample concentration was standardized by mass of the pulverized eggshell sample.

### 2.3 DNA extraction and 16S sequencing

We combined 0.150 g of pulverized eggshell from the top half of an egg and 0.150 g of pulverized eggshell from the bottom half of the same egg. DNA was extracted from the combined 0.300 g eggshell fragments using the PowerSoil DNA Isolation kit (Mo Bio Laboratories, Carlsbad, California, USA). Homogenization of the pulverized eggshell was conducted with the kit's PowerBead tubes mounted in a vortexer per the kit's instructions. Ultimately, purified DNA was eluted in 100 μl of buffer C6 from the PowerSoil kit.

We confirmed the success of DNA extractions by PCR with primers 515F and 806R which amplify the V4 variable region of the 16S rRNA of bacteria (Caporaso et al., 2011). Reaction components were 1.5 μl 10× GeneAmp buffer, 1.2 μl 25 mmol/L MgCl_2_, 1.5 μl 2 mmol/L dNTP, 0.4 μl 10 μmol/L primer 515F, 0.4 μl 10 μmol/L primer 806R, 6.175 μl water, 0.075 μl AmpliTaq Gold DNA polymerase, and 3.75 μl template DNA. Cycling parameters were 95°C for 10 min, followed by 32 cycles of 95°C for 45 s, 55°C for 60 s, and 72°C for 90 s, ending with 72°C for 10 min and a 4°C hold. Negative control PCRs confirmed a lack of contamination in our lab workflow.

After this PCR amplification in our own lab had verified the presence of an amplicon of the expected 300 bp size in all samples, the set of extracted DNA samples was sent to a commercial lab (MR DNA, Shallowater, Texas, USA) for PCR and high‐throughput sequencing of the V4 variable region of the 16S rRNA gene. This commercial PCR was conducted with barcoded versions of primers 515F and 806R in a single‐step 30 cycle reaction using HotStarTaq Plus Master Mix Kit (Qiagen, USA) with the following cycle parameters: 94°C for 3 min; followed by 28 cycles of 94°C for 30 s, 53°C for 40 s and 72°C for 1 min; followed by a final elongation step at 72°C for 5 min. Sequencing was performed on an Ion Torrent PGM following the manufacturer's guidelines, with data subsequently processed by the commercial lab using a proprietary analysis pipeline (MR DNA, Shallowater, Texas).

Sequences were depleted of barcodes and primers, then filtered to exclude sequences that were <150 bp, had ambiguous base calls, or had homopolymer runs exceeding 6 bp. After denoising and chimera removal, the remaining sequences were taxonomically classified using BLASTn against a curated GreenGenes database (Desantis et al., 2006). For each eggshell sample, data were expressed as the relative percentage of sequences within each sample that map to the designated taxonomic classification. This allows comparison of community makeup across eggs while standardizing for differences between eggs in overall efficiency of DNA extraction or amplification.

### 2.4 Repeatability

To assess repeatability of our bacteria characterization, we repeat‐assayed 13 eggs by independent DNA extraction, amplification, and sequencing of duplicate subsamples of the pulverized eggshell. We measured the repeatability of eggs’ percent Gram‐positive sequences, Bray–Curtis dissimilarities at the level of orders, and Principal Component scores of relative abundance based on the nine most abundant orders, by computing the intraclass correlation coefficient (ICC; Lessells & Boag, 1987). For all other downstream analyses, the duplicate characterizations of a given eggshell were averaged.

### 2.5 Community characterization and data analysis

Our first aim was a field‐based experimental test of the hypothesis that the brown eggshell pigment protoporphyrin has photodynamic antimicrobial activity against Gram‐positive bacteria (Ishikawa et al., 2010). We predicted that the bacteria community of brown eggs should comprise proportionally fewer Gram‐positive bacteria above ground where they were exposed to light than underground in the dark burrows of storm‐petrels; however, we predicted no such effect in white eggs that lack protoporphyrin. We tested this prediction using a linear mixed model to ask whether the proportion of Gram‐positive bacteria varied by sunlight exposure (in a burrow or above ground), eggshell color (either brown or white), the interaction between sunlight exposure and eggshell color, and date (categorical: June 16 or June 18 deployment). This model included site as a random effect to benefit from the matched nature of our experimental design (Figure 1). We transformed (arcsine‐square root), centered, and scaled the proportion of Gram‐positive bacteria in these analyses and retained nonsignificant interactions in our model as these are essential to our experimental design (Schielzeth & Forstmeier, 2009). Whole model significance for linear mixed models was established via likelihood ratio tests comparing each model to similarly constructed null models including only an intercept. These models were fit via maximum likelihood. The significance of fixed effects was calculated via Wald χ^2^ tests, and we report two *r* ^2^ values for linear mixed models (Nakagawa & Schielzeth, 2013). One evaluates the variance explained by the fixed effects alone (marginal *r* ^2^, hereafter $r_{m}^{2}$), while the second represents the variance explained by the entire model, that is, including both the fixed and random effects (conditional *r* ^2^, hereafter $r_{c}^{2}$). All parameter estimates and data are presented as mean ± *SE*, and all statistical analyses were conducted in R version 3.3 (R Development Core Team 2016).

Our second aim was to confirm whether our sample sizes and methods allowed us to describe any biologically meaningful variation in avian eggshell bacterial composition. Accordingly, we set out to more broadly assess whether and how bacteria communities on eggshells vary with eggshell color and between two nesting environments—on the surface of the ground versus below ground in a storm‐petrel burrow. For these analyses, we aimed to compare eggs in their bacteria community composition; that is, the relative abundance of different bacterial taxa, rather than simply Gram‐positive versus Gram‐negative bacteria. Choosing the taxonomic level to use for community characterization entails a tradeoff about granularity: a very coarse scale (e.g., phyla) can treat as equivalent those bacteria types that are actually very different from each other (Philippot et al., 2010), but a very fine scale (e.g., species) yields an unmanageable number of taxa and many zeroes for abundance values. To strike a balance, we examined diversity at the taxonomic level of orders, which is likely to maintain a signature of community structure (Philippot et al., 2010).

Our sequences fell into 115 orders. To compare the beta diversity of order‐level bacteria community composition of eggshells, we used two complementary approaches: the Bray–Curtis dissimilarity index (Birtel, Walser, Pichon, Bürgmann, & Matthews, 2015), and Principal Components Analysis (PCA). For calculating Bray–Curtis dissimilarity between eggs, we retained the relative abundance data from all 115 orders, because the Bray–Curtis index is not heavily influenced by extremely rare taxa (Krebs, 1999). Bray–Curtis dissimilarity is defined as

$${\text{BC}}_{j,k}=\frac{\sum_{i=1}^{n}\left|X_{ij}-X_{ik}\right|}{\sum_{i=1}^{n}\left(X_{ij}+X_{ik}\right)}$$

where *j* and *k* are the two eggshells being compared, *n *= the number of bacteria taxa found in those two eggshells, and *X*_*ij*_ and *X*_*ik*_ are the percentage of an eggshell's bacteria sequences that belong to taxon *i* in samples *j* and *k*, respectively. We calculated Bray‐Curtis dissimilarities between all pairwise comparisons of eggs using the vegan package in R (Oksanen et al., 2016). We used a linear model to test whether the resulting pairwise Bray‐Curtis dissimilarities describing eggs’ bacteria communities were more similar if the eggs were the same color, were exposed to the same type of location (above ground or in a burrow), or were exposed to the environment on the same date.

In addition, we used a Principal Component Analysis (PCA) to examine the variation in the community structure of bacteria on eggshell surfaces. To ensure a reasonable ratio of subjects‐to‐variables (Grimm & Yarnold, 1995), we truncated our data to bacteria orders that had a median greater than or equal to 2% of each egg's sequences. This elimination of rare taxa yielded a dataset of nine orders for PCA (Table 1), in which each egg retained 48.3% to 85.7% (median 65.3%) of its initial bacterial sequences. This PCA was based on the covariance matrix, as all these data were of the same units and scale, and yielded four Principal Components with eigenvalues greater than 1 (see Section 3). Next, we used four separate linear mixed models to test whether eggs’ scores on each of the four Principal Components varied systematically with egg color, sunlight exposure (above ground versus burrow), or date. The specification of these linear mixed models was the same as in the analysis of percentage of Gram‐positive sequences, and each model controlled for site as a random effect.

**Table 1:** Factor loadings from Principal Component Analysis of relative bacterial abundance data from the nine most common bacteria orders (*n* = 44 eggs). Loadings > |0.5| are shown in bold

Order	PC1 (55%)	PC2 (22%)	PC3 (12%)	PC4 (6%)
Pseudomonadales	**0.908**	0.223	0.170	−0.211
Actinomycetales	−0.111	−0.093	−0.274	−0.026
Lactobacillales	0.069	**−0.638**	**0.601**	0.380
Clostridiales	−0.097	−0.472	0.016	**−0.857**
Rhizobiales	−0.078	0.074	−0.115	0.103
Burkholderiales	−0.051	0.073	−0.228	0.012
Legionellales	−0.372	**0.539**	**0.675**	−0.243
Xanthomonadales	−0.028	0.049	−0.079	0.081
Sphingobacteriales	−0.037	0.091	−0.084	0.014

## 3 RESULTS

### 3.1 Protoporphyrin content

Pigment analysis confirmed that the color difference between brown and white eggs was indicative of eggshell pigment content: all brown eggshells contained detectable and quantifiable levels of protoporphyrin (1.2 ± 0.09 μmol L^−1^ g^−1^; range 0.68 to 1.78 μmol L^−1^g^−1^), whereas the white eggshells contained no trace of this pigment. Neither white nor brown eggs contained detectable biliverdin concentrations.

### 3.2 Bacterial community characterization

After filtering out sequences that were incomplete, ambiguous, or had long homopolymer runs, the median read depth per eggshell sample was 28,527 valid sequences (range 5,955 to 338,990). After combining the duplicate sequencing results from the 13 eggshells that were replicated, the median read depth per eggshell was 47,651 valid sequences (range 5,955 to 338,990).

Across 44 eggs, the proportion of reads that were Gram positive ranged from 10.3% to 78.6% (median 32.6%). Overall, the most commonly represented bacteria orders were Pseudomonadales (Gram negative, motile by polar flagella; median 16.8%, max 63.2%), Actinomycetales (Gram positive, aerobic, sporulating; median 7.5%, max 17.0%), Lactobacillales (Gram‐positive, acid tolerant, low G+C content in their DNA; median 5.6%, max 36.5%), Clostridiales (Gram positive, low G+C, anaerobic; median 4.5%, max 38.3%), and Rhizobiales (Gram negative, nitrogen‐fixing symbionts; median 4.1%, max 20.6%) (see data archive). At the genus level, the most common taxa were *Pseudomonas* (median 5.3%, max 57.5%), *Lactobacillus* (median 1.8%, max 23.1%), *Rickettsiella* (median 1.8%, max 52.3%), and *Acinetobacter* (median 1.5%, max 30.2%).

### 3.3 Test of photodynamic activity of protoporphyrin against Gram positive bacteria

**Figure 2:**
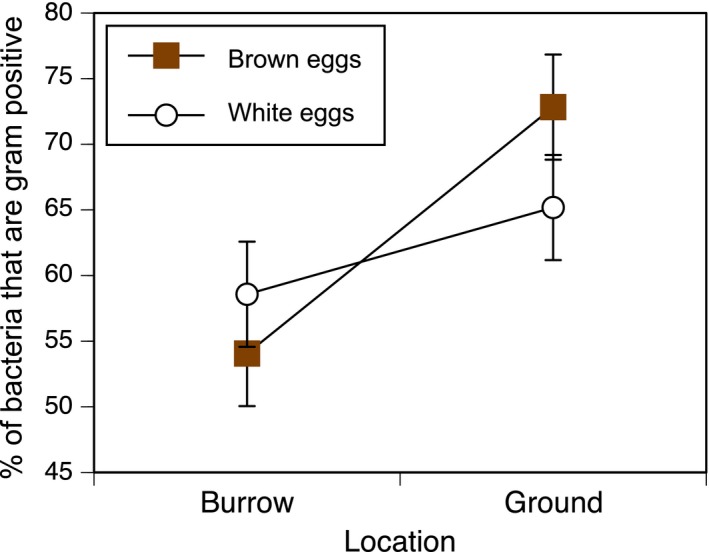
Percent of bacteria that are Gram positive, as a function of location type (in a burrow versus exposed to sunlight above ground) and whether eggs are brown (filled symbols) or white (open symbols). The hypothesis of photodynamic antimicrobial activity of protoporphyrin predicts an interaction between nest type and egg color, with proportionally fewer Gram positives on brown eggs when exposed to sunlight. The model's interaction term was not significant (see Section 3); moreover the suggestion of a trend is in the unpredicted direction. Values shown are marginal means ± *SE*, accounting for an effect of deployment date

The proportion of the sequencing reads that mapped to Gram‐positive bacteria was tested in a model containing the predictor variables of eggshell color, site, the interaction between these two factors, and the random effect of site (χ^2^ = 18.4, $r_{m}^{2}=0.35$, $r_{c}^{2}=0.39$, *p* = .001). However, in this overall model, neither the color‐by‐sunlight interaction ($\chi_{1}^{2}=2.77$, β = −0.77 ± 0.49, *p* = .10; Figure 2) nor main effect of eggshell color ($\chi_{1}^{2}=0.76$, β = 0.29 ± 0.35, *p* = .38) was significant. In contrast, there was a main effect of date ($\chi_{1}^{2}=8.13$, β = −0.75 ± 0.29, *p* = .004) such that there were proportionally more Gram‐positive bacteria on the first deployment date, and there was a main effect of location type ($\chi_{1}^{2}=13.17$, β = 1.20 ± 0.35, *p* < .001) such that proportionally more Gram‐positive sequences were detected on eggs exposed to sunlight than on eggs placed in dark burrows.

### 3.4 Comparisons of bacteria community by egg color, location, and sampling date

In a linear model analyzing the pairwise Bray‐Curtis dissimilarity measures of beta diversity between eggs, based on sequences from all 115 detected bacteria orders, the eggs’ bacteria communities were more similar if they were both above ground or both in burrows (*F*_1,942_ = 73.5, *p* < .0001) and if they were exposed to the environment on the same date (*F*_1,942_ = 5.7, *p* = .017); in contrast, being the same color (i.e., both brown, or both white) did not lead to eggs being more similar in their bacteria communities (*F*_1,942_ = 0.1, *p* = .741). Despite the significant effects of location type and date, the overall model explained little of the variation in Bray‐Curtis dissimilarities (*F*_3,942_ = 26.1, *p* < .0001, Adjusted *R* ^2^ = 0.074).

As a complement to Bray‐Curtis dissimilarities, we also condensed the abundance data from the nine most common orders into four Principal Components, which captured 55%, 22%, 12%, and 6% of the variance (95% total) in the nine original variables (Table 1). Subsequent linear mixed models to explain variation across eggs in each of the four PC scores found predictors for only the first two PCs, as detailed below.

**Figure 3:**
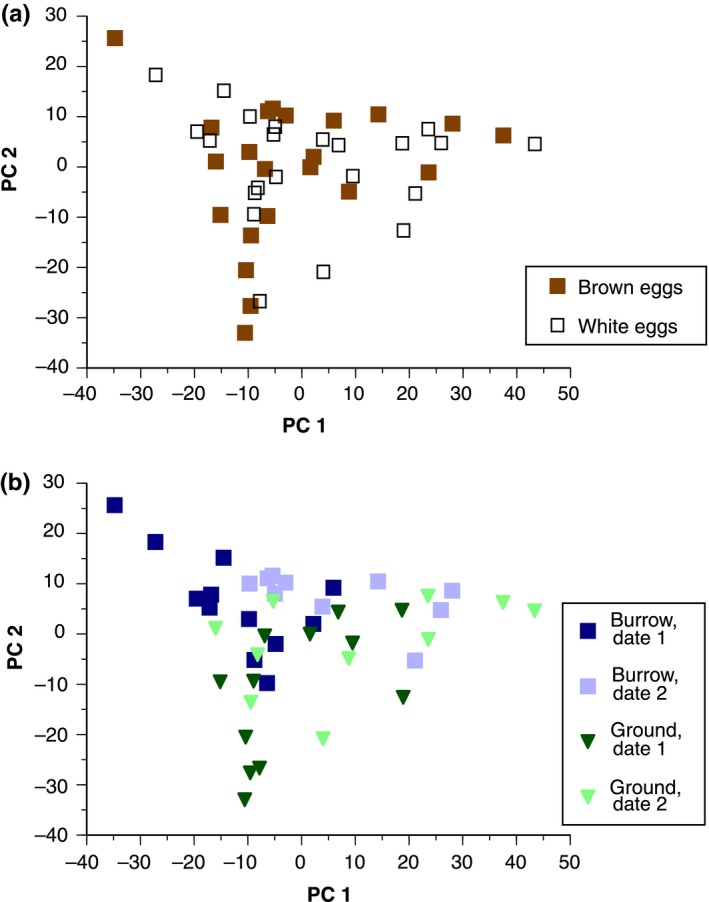
Principal Component (PC) scores on relative bacterial abundance data from 44 eggs. (a) Brown eggs did not differ from white eggs along PC1 or PC2. (b) Burrow eggs (square symbols) scored higher than ground eggs (triangles) along PC2, while the two deployment dates (dark versus light symbols) differed along PC1

PC1 had a strong positive loading of Pseudomonadales, and variation between eggs in PC1 was significantly predicted by our linear mixed model (χ^2^ = 13.82, $r_{m}^{2}=0.31$; $r_{c}^{2}=0.33$, *p* = .008; Figure 3). In that model, PC1 was significantly explained by deployment date (χ^2^ = 11.71, β = 15.18 ± 4.90, *p* < .001) such that PC1 scores were larger on the second deployment. By contrast, PC1 was not significantly explained by eggshell color (χ^2^ = 0.14, β = −2.14 ± 6.11, *p* = .71), or whether eggs were on the ground versus in burrows (χ^2^ = 0.16, β = 2.32 ± 6.11, *p* = .69), or an interaction between color and sunlight exposure (χ^2^ = 1.90, β = 11.34 ± 8.64, *p* = .17).

PC2 had moderate negative loadings of Lactobacillales and Clostridiales and a positive loading of Legionellales, and variation between eggs in PC2 was significantly predicted by a linear mixed model (χ^2^ = 22.81, $r_{m}^{2}=0.39$; $r_{c}^{2}=0.48$, *p* < .0001; Figure 3). In this model, PC2 was significantly predicted by whether eggs were on the ground versus in burrows (χ^2^ = 21.76, β = −17.58 ± 3.95, *p* < .0001), such that eggs kept in burrows had larger PC2 scores. In contrast, PC2 was not predicted by eggshell color (χ^2^ = 0.47, β = −2.57 ± 3.59, *p* = .49), an interaction between color and sunlight exposure (χ^2^ = 2.04, β = 7.61 ± 5.59, *p* = .15), or date of deployment (χ^2^ = 2.21, β = 5.15 ± 3.83, *p* = .14).

### 3.5 Repeatability

Based on repeat assays of DNA extraction, PCR amplification, and high‐throughput sequencing, all measures of eggshell bacteria communities were significantly repeatable. Repeatability was high for the crucial metric of the percent of sequences belonging to Gram‐positive taxa (intraclass correlation coefficient ICC = 0.778, *F*_12,24_ = 8.02, *p* = .0005). Repeatability was moderate to high for our other descriptive measures, including Principal Component scores on all four retained components on order‐level relative abundance (PC1: ICC = 0.722, *F*_12,13_ = 6.2, *p* = .001; PC2: ICC = 0.800, *F*_12,13_ = 9.0, *p* = .0002; PC3: ICC = 0.496, *F*_12,13_ = 3.0, *p* = .031; PC4: ICC = 0.825, *F*_12,13_ = 10.4, *p* < .0001) and Bray‐Curtis measures of dissimilarity between eggshells (ICC = 0.336, *F*_77,78_ = 2.01, *p* = .001).

## 4 DISCUSSION

The primary aim of our study was to use a field experiment to test the antimicrobial hypothesis, that the avian eggshell pigment protoporphyrin has a photodynamic antimicrobial function against Gram‐positive bacteria. Our experiment examined matched sets of brown versus white domestic chicken eggs deployed under natural conditions in the field and did not support the antimicrobial hypothesis. Despite data confirming that all brown eggs had measurable concentrations of protoporphyrin relative to the pigment‐free white eggs, neither egg color nor the interaction between color and light exposure predicted the proportion of Gram‐positive bacteria harbored by the eggshells in our studies. Thus, our study does not support an evolutionary function of avian eggshell pigmentation as an inhibitor of bacterial survival.

Although we did not find the predicted interaction between egg color and location, our data were of sufficient quality to detect other patterns. In particular, we found strong repeatability for all metrics of our sequencing outputs, including the percent of Gram‐positive bacteria found on individual eggs (ICC = 0.778), and we detected diverse bacteria communities that varied reliably between two biologically meaningful factors: temporal variation (deployment date) and location type (above or below ground). Temporal variation, as implicated by date of egg deployment, was a predictor of Gram‐positive percentages and overall order‐level beta diversity across bacteria communities (whether measured with Bray‐Curtis dissimilarity or PCA). Our dates of deployment did not differ substantially in ambient temperature (temperature ranges of 9 to 18°C and 9 to 20°C, respectively), but there was a significant rain event during the first deployment period. Rain might alter opportunities for bacteria to colonize and persist on eggs above the ground, by allowing soil bacteria to move onto the eggshell through bouncing water droplets or perhaps by having rainfall physically dislodge existing colonies from an eggshell. In addition to these temporal patterns, we found differences in bacterial community assemblages on eggshells that had been deployed in above‐ground sites versus below‐ground sites. These location‐based differences could stem from differences in what bacteria exist there, including bacteria from feathers or feces of storm‐petrels breeding in the burrows in previous years. These differences could also stem from the growing conditions in the two types of locations, as the underground burrows have higher humidity and more stable temperatures, which might favor the proliferation of a different subset of bacteria than at the above‐ground sites.

Eggs in our study design were deliberately not incubated, but incubation behavior is another factor that can influence eggshells’ microbial communities, with potential impacts on the risk of trans‐shell infection and the resulting viability of the embryo (Cook, Beissinger, Toranzos, & Arendt, 2005). Incubation by an adult bird can alter eggshell bacteria communities by facilitating desiccation (D'alba, Oborn, & Shawkey, 2010), increasing eggshell temperatures (Grizard et al., 2014), transferring antimicrobial secretions from the uropygial gland (D'alba & Shawkey, 2015), and blocking the sunlight needed for photodynamic antimicrobial effects of protoporphyrin (Ishikawa et al., 2010). Our experiment did not address these possible effects of incubation behavior on microbial communities, but, instead, simulated the part of the laying period when eggs are often left unattended for one or more days prior to the onset of full incubation (Hebert, 2002; Wang & Beissinger, 2009). We chose a two‐day period, which is within the wide range of incubation‐onset latencies recorded in various bird species (Stoleson & Beissinger, 1995). An important question is whether enough microbial growth would occur over this two‐day period to provide a strong test of the antimicrobial hypothesis. This issue is worthy of discussion (see below) and, particularly, worthy of exploration in future experiments.

Studies in tropical settings have clearly shown the potential for rapid bacterial growth on avian eggshells (Cook, Beissinger, Toranzos, Rodriguez, & Arendt, 2003; Cook, Beissinger, Toranzos, & Rodriguez et al., 2005), but data from temperate settings are limited and conflicting. On one hand, Wang, Firestone, and Beissinger (2011) found that bacteria loads on eggshells did not increase with duration of exposure in unattended nestboxes in a dry, temperate‐zone study site. On the other hand, in a more humid temperate‐zone experiment by Godard et al. (2007), in which eggs were regularly misted with water, bacteria loads on eggshells did increase over time. Although those two studies differed in many respects, moisture might be the key difference affecting bacteria loads (Cook, Beissinger, Toranzos, & Rodriguez, 2005), in which case our design more closely resembles Godard and colleagues’, as our all of our eggs were exposed to rain, fog, or extremely high humidity (>90%). Thus, we consider that conditions in our study favored bacterial growth sufficiently to have made a meaningful test of the antimicrobial hypothesis for protoporphyrin. Furthermore, our study did find biologically detectable patterns of variation in bacteria community composition, including a difference between burrow‐deployed eggs and ground‐deployed eggs in the proportion of bacteria that were Gram positive. Regardless, it is reasonable to imagine that bacterial growth rates and avian incubation onset patterns differ between study systems in a way that translates into different strengths of selection on the potential antimicrobial properties of protoporphyrin. Future experiments could use a longer deployment time (i.e., to simulate a less typical bird species which has a longer delay between laying and incubation) or could conduct the experiment in a study area with a warmer climate that naturally facilitates faster bacteria growth (Cook, Beissinger, Toranzos, & Arendt, 2005).

Another factor to explore is the type of egg used in such experiments: commercial versus wild‐sourced, solid versus maculated, and with protoporphyrin in the calcareous layer versus in the cuticle. We address these points briefly in turn. First, we chose commercially sourced eggs to standardize as many variables as possible, including having both brown and white eggs from the same species. Eggs from wild birds might show within‐population variation in protoporphyrin and thus in the scope for antimicrobial activity; this could be a fruitful line of questioning eventually, but the use of wild‐sourced eggs in initial experiments such as ours would likely lead to reduced power for detecting a fundamental difference in bacteria communities on eggs with and without protoporphyrin. Second, we used solidly pigmented eggs, as was performed in the lab experiments that motivated our study (Ishikawa et al., 2010), to maximize our power by ensuring that there was protoporphyrin present in the regions of the shell that we were sampling for bacteria. If a similar experiment were conducted with maculated shells, only a subset of the shell's surface would be subject to any antimicrobial effects of protoporphyrin, and it would be challenging but intriguing to try to assay microbes separately on the spotted and unspotted parts of the same egg. Third, protoporphyrin in brown eggs of chickens (our study) is deposited primarily in the calcareous layer of the shell rather than mainly in the cuticle (Samiullah & Roberts, 2013). This was one factor in our decision to sample bacteria by eggshell crushing rather than by simply swabbing the outermost surface. In systems where protoporphyrin is found mainly in the cuticle (Fargallo et al., 2014), the cuticular pigment can be removed experimentally from freshly laid eggs; although one experiment has found no effect of protoporphyrin removal on embryo viability or post‐hatching survival (Fargallo et al., 2014), it would be interesting to test whether removing protoporphyrin causes the predicted increase in Gram‐positive bacteria on the eggshell.

Overall, our experiment did not find support for a photodynamic antimicrobial activity of protoporphyrin. To further assess the generality and importance of this finding, we recommend replicating some of our methodological approaches in future work that examines the combined role of eggshell pigments with physical and biological factors that could influence the antimicrobial defense mechanisms of bird eggs and the intrinsic scope for bacterial colonization and growth on eggshells. Such factors could include natural and nest‐specific humidity, nest material composition, and environmental and nest‐specific temperatures. All of these could affect the intrinsic growth of bacteria on eggshells, the effectiveness of antimicrobial defenses, and, ultimately, embryonic viability due to trans‐shell infections (Brandl et al., 2014; Cook et al., 2003; D'Alba et al., 2016; Ruiz‐Castellano, Tomás, Ruiz‐Rodríguez, Martín‐Gálvez, & Soler, 2016).

## CONFLICT OF INTEREST

The authors declare no conflict of interest.

## DATA ACCESSIBILITY

Data available from the Dryad Digital Repository: https://doi.org/10.5061/dryad.s1n6p.

## AUTHOR CONTRIBUTIONS

DCD designed the study. Field work and lab work were conducted by DCD, SP, and MD. DH, DCD, and SP analyzed data. DCD, DH, MD, and MEH drafted the paper, and all authors helped with revisions.
